# Application of Ultrasound in Detecting and Removing Migrating Grass Awns in Dogs and Cats: A Systematic Review

**DOI:** 10.3390/ani13132071

**Published:** 2023-06-22

**Authors:** Domenico Caivano, Francesca Corda, Andrea Corda, Giulia Moretti, Antonello Bufalari

**Affiliations:** 1 Department of Veterinary Medicine, University of Perugia, Via San Costanzo 4, 06126 Perugia, Italy; domenico.caivano@unipg.it (D.C.); antonello.bufalari@unipg.it (A.B.); 2 Department of Veterinary Medicine, University of Sassari, Via Vienna 2, 07100 Sassari, Italy; francescacorda91@tiscali.it

**Keywords:** foreign body, ultrasonography, grass awn, canine, feline

## Abstract

**Simple Summary:**

The aim of this systematic review was to summarize the current knowledge on the application of ultrasonography in the identification and removal of grass awns from various anatomic locations in dogs and cats. We selected and analyzed 46 papers on the application of ultrasonography in dogs and cats affected by migrating grass awns. The ultrasonographic appearance of grass awns is characteristic, although their size and location can influence visualization and removal attempt. In some cases, migrating grass awns are not directly visualized by ultrasonography, but lesions caused by their migration can be easily seen. Ultrasonography can be considered a useful diagnostic tool to localize and remove migrating grass awns; however, when the migration occurs in less accessible locations or discrete foreign bodies are present, this diagnostic tool should be considered as a part of a multidisciplinary approach with advanced diagnostic imaging modalities.

**Abstract:**

Migrating grass awns are an important cause of disease in dogs and cats. Plant awns can migrate into several body tissues and cavities because of their fusiform shape and backward-pointing barbs. Their migration causes inflammatory tissue reaction and clinical signs depend upon their localization. Ultrasound has been described as a useful, noninvasive, and readily available tool to identify and guide vegetal foreign bodies removal in animals. The aim of this systematic review was to summarize current knowledge on the application of ultrasonography in the identification and removal of grass awns from various anatomic locations in dogs and cats. We selected and analyzed 46 papers on the application of ultrasonography in dogs and cats affected by migrating grass awns. The ultrasonographic appearance of grass awns is characteristic, although their size and location can influence the visualization and the attempt of removal. In some cases, migrating grass awns are not directly visualized by ultrasonography, but the lesions caused by their migration can be easily seen. Ultrasonography can be considered a useful diagnostic tool to localize and remove migrating grass awns; however, when the migration occurs in less accessible locations or discrete foreign bodies are present, this diagnostic tool should be considered as a part of a multidisciplinary approach with advanced diagnostic imaging modalities.

## 1. Introduction

**Figure 1:**
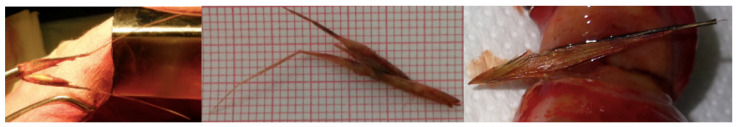
Grass awns removed from various anatomic locations with characteristic fusiform shape.

Migrating grass awns are an important cause of disease in companion animals. Plant awns can migrate into several body tissues and cavities because of their fusiform shape and backward-pointing barbs (Figure 1). Their migration causes inflammatory tissue reaction and clinical signs depend upon their localization. Medical treatment with antimicrobial and anti-inflammatory drugs can initially allow a clinical improvement but removal of the foreign body is essential for the resolution of this pathological condition.

The visualization and exact localization of the grass awn increases the success rate during the removal attempt. Imaging techniques such as fistulography, radiography, ultrasonography, computed tomography, and MRI have been used to identify and guide the removal of migrating grass awns in dogs and cats [1,2,3,4,5,6,7]. Ultrasound (US) has been described as a useful, noninvasive, and readily available tool to identify and guide vegetal foreign bodies removal in animals [8]. Moreover, preoperative US in combination with intraoperative US demonstrated to be useful in removing migrating grass awns in various tissues, reducing the risk of their fragmentation or iatrogenic damage [9,10].

The aim of this systematic review was to summarize the current knowledge on the application of US in the identification and US-guided removal of grass awns from various anatomic locations in dogs and cats.

## 2. Materials and Methods

This review was carried out according to the Preferred Reporting Items for Systematic Reviews and Meta-Analyses (PRISMA) guidelines. A literature search was conducted using PubMed and Scopus databases. Only peer reviewed papers written in English language concerning the application of US in the identification and removal of grass awns in dogs and cats were included. Published literature until 31 December 2022 was searched using the following key words:

Ultrasonography (ultrasound) AND vegetal foreign body;
Ultrasonography (ultrasound) AND grass awn;
Ultrasonography (ultrasound) AND removal AND vegetal foreign body;
Ultrasonography (ultrasound) AND removal AND grass awn;
Ultrasonography (ultrasound) AND plant foreign body AND veterinary.

**Figure 2:**
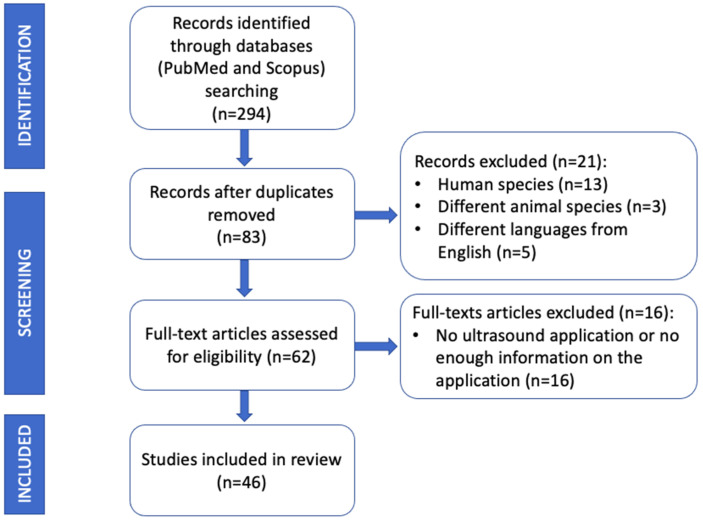
Modified Preferred Reporting Items for Systematic Reviews and Meta-Analyses (PRISMA) flow diagram with the review search strategy and study selection.

All papers in which US was not performed in the diagnostic process or concerning different species from dogs and cats were excluded from the review. Conference abstracts were also excluded. Article abstracts were exported into a reference manager software (Mendeley Desktop version 1.19.8) and duplicates were removed (Figure 2).

## 3. Results

A total of 46 papers met the inclusion criteria. The selected publications described the application of US in detecting and removing migrating grass awns located in various body tissues and cavities of dogs and cats. Based on the anatomical localization of the grass awns, the papers were grouped:

1. Intrathoracic migration (10 papers);
2. Retroperitoneal migration (13 papers)
3. Intraabdominal migration (6 papers);
4. Periocular migration (8 papers);
5. Genitourinary tract migration (8 papers);
6. Subcutaneous tissue migration (8 papers).

### 3.1. Intrathoracic Migration

**Figure 3:**
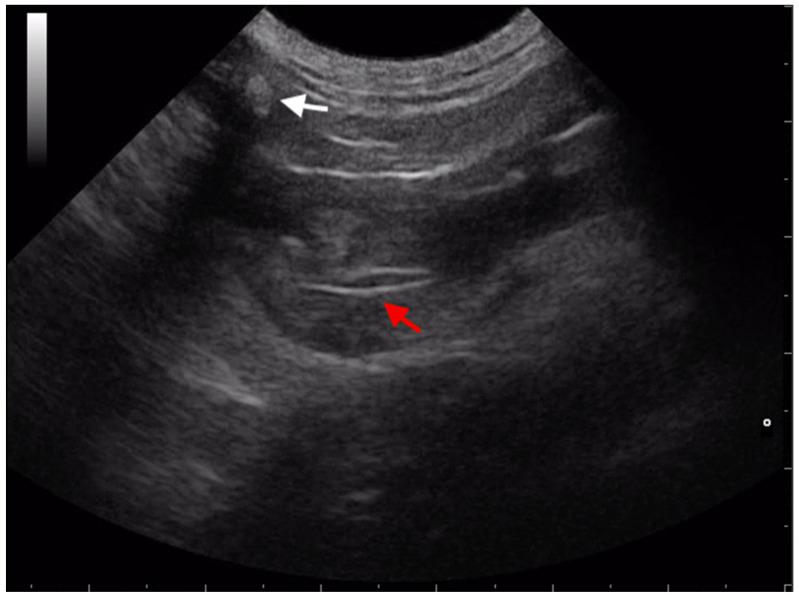
Transthoracic ultrasonographic image of a spindle-shaped hyperechoic foreign body consistent with a migrating grass awn (red arrow) in the pleural space of a dog. White arrow indicates a rib.

Inhalation of grass awns commonly cause respiratory disorders in dogs and cats, mostly during spring and summer seasons. The prevalence of respiratory disease, secondary to grass awns intrathoracic migration, is higher in young hunting and working dogs probably because of increased exposure to plants in the field while exercising and open-mouth breathing [4]. Clinical signs can go unnoticed by the owners or disappear after treatment with antibiotic/anti-inflammatory drugs, resulting in subsequent migration through the airways. It has been theorized that the inhaled grass awns travel down the trachea and the caudal bronchi and penetrate the lungs, pleural, and/or pericardial space, thoracic or abdominal wall, or into the abdominal or retroperitoneal cavity [2,11,12]. Migration of plant material introduce bacteria, incite a foreign body response, interfere with local host defenses, and provide a nidus for chronic infections, frequently manifesting as abscesses and draining tracts [2,11,13,14]. Intrathoracic grass awn migration can cause focal pneumonia, pleural, and/or pericardial effusion and thickening, mediastinal abscessation, pneumothorax, or a combination of these conditions in dogs [4,9,12,15,16,17]. The most common presenting compliant of intrathoracic grass awn migration are cough and hyperthermia [4]. The first report that described the ultrasonographic findings related to intrathoracic grass awns migration in dogs and cats was published by Schultz et al. in 2008. The main ultrasonographic findings, described in eight animals, were pleural effusion, lung lobe consolidation, complex soft tissue mass, and foreign body identification [4]. Subsequently, Caivano et al. described a case report in which transesophageal ultrasonography was used, for the first time, to localize an intrapulmonary grass awn foreign body in an English Setter dog. The foreign body appeared as a linear hyperechoic structure within an area of pulmonary consolidation in the dorsomedial portion of the left lung. Transesophageal ultrasonography provided important information for the subsequent thoracoscopic removal [16]. The medial portions of the lungs are not visualized by transthoracic US; therefore, foreign bodies and related lesions located in these areas, and adjacent to the esophagus, can only be visualized using transesophageal probes. However, as acknowledged by the same authors, transesophageal ultrasonography requires general anesthesia and the use of specialized and expensive probes [16]. In 2016, the same group of researchers described the ultrasonographic findings of 43 dogs with suspected intrathoracic grass awns. In this retrospective study, the authors scanned the thoracic structures through transthoracic ultrasonography in all 43 dogs, transesophageal ultrasonography in 14 of them, and intraoperative ultrasonography in 11 of them. Grass awns were identified by transthoracic US in 20 dogs (47%): awns were localized to the pleural space and pulmonary parenchyma of 13 and 7 dogs, respectively. Transesophageal ultrasonography was able to identify and localize vegetal foreign bodies in the medial pulmonary parenchyma, close to the esophagus, of 3 dogs. Grass awns generally appeared as spindle-shaped hyperechoic structures of variable length (range, 0.8 to 2.5 cm) often surrounded by a hypoechoic zone, consistent with a focal inflammatory response (Figure 3). In dogs with migrating pleural grass awns, pleural effusion, pleural thickening, and enlargement of sternal lymph nodes were present and the vegetal foreign bodies were observed in the ventral region of the thorax, entrapped in a pleural abscess or granuloma or in the thickened pleura. In dogs with migrating pulmonary grass awns areas of pulmonary consolidation, focal lung adherences and focal pleural effusion were visualized. The authors of this study highlight that pleural effusion and/or lung consolidation, which frequently surrounds intrathoracic grass awns, can provide an acoustic window that increases the ability to examine intrathoracic lesions and to identify foreign bodies. This paper provided evidence of the utility of ultrasonography for diagnosis, localization, and intraoperative guidance for surgical removal of suspected migrating intrathoracic grass awns in dogs [9]. In 2018, Shan and Ruth reported a case of pulmonary granuloma and vertebral spondylitis secondary to inhalation of grass awn in a dog. In this case report, transthoracic US identified the lung lesion but failed to visualize the foreign body within it. Authors speculated that ultrasonography was not informative because the foreign body was in a gas-filled bronchus [18]. In a large retrospective study published by Philp et al. in 2020, US was used to search for intrathoracic plant foreign bodies and enabled localization, although no accuracy in the identification was reported [13]. Vegetal foreign bodies are less frequently reported in cats compared with dogs. Cola et al. described the use of ultrasonography in detecting thoracic grass awns in 4 cats presented for respiratory signs [19]. Ultrasound examination evidenced unilateral pleural effusion and atelectasis of the ventral portion of the lung lobes of the affected side in all the cases. In one cat, a mediastinal lanceolate binary structure characterized by two hyperechogenic walls separated by a central hypoechogenic band, compatible with a plant foreign body, was visualized during the first US scan. In the other 3 cats, 3 to 5 US examinations repeated on different days were required to identify the foreign bodies in the mediastinum and pleural space [19]. Migrating intrathoracic grass awns can rarely localize in the pericardium. Two papers, one on a dog and one on a cat, reported septic pericarditis caused by a migrating grass awn visualized by US [15]. In both papers, under ultrasonographic guidance sternotomy enabled the removal of migrating grass awn from the pericardium [15,20].

### 3.2. Retroperitoneal Migration

**Figure 4:**
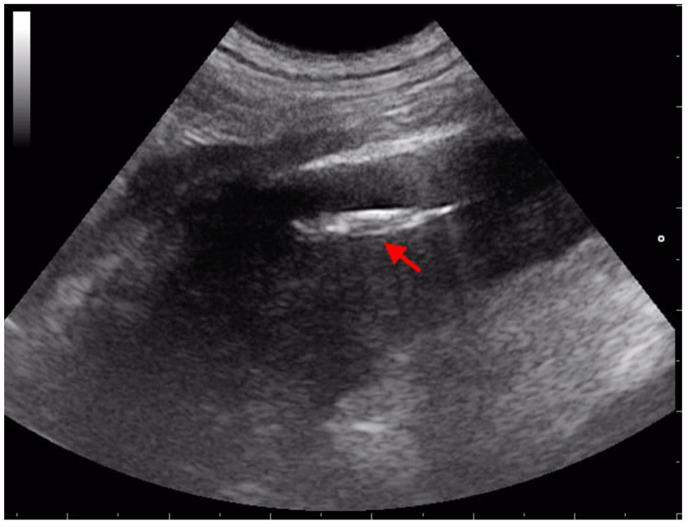
Transabdominal ultrasonographic image of a spindle-shaped hyperechoic foreign body consistent with a migrating grass awn (red arrow) in the left iliopsoas muscle of a dog.

Retroperitoneal grass awn migration in companion animals commonly causes local inflammation and septic tissue reaction associated with flank swelling, back or abdominal pain, pyrexia, and depression [2,5,21]. Different routes of entry of plant material into the retroperitoneal region have been proposed. Some authors speculated that grass awns, after being inhaled and having migrated into the thoracic cavity, can penetrate the diaphragm reaching the retroperitoneum [2,21,22]. This theory is strengthened by the fact that prodromal respiratory symptoms are reported in many cases, no skin lesions are observed, and hunting dogs are predominantly affected [2,7,23]. On the contrary, other authors hypothesized that the vegetal foreign bodies arrived in the retroperitoneal region after ingestion and penetration of the intestinal wall or by penetration of the skin [24]. Several papers described the visualization of grass awns in the iliopsoas muscles (Figure 4). This localization seems to be frequent in dogs [10,25,26,27]. In 1999, Frendin et al. demonstrated the utility of US in detecting lesions of the iliopsoas muscles in five hunting dogs with back pain. Ultrasonography showed increased diameter and abnormal echogenicity of sublumbar muscles in all the patients, and in three of them, revealed the presence of a hyperechoic structure compatible with a vegetal foreign body. In this paper, grass awns were surgically removed in all the dogs [2]. An Australian study, published in 2004 by Staudte and colleagues, reported the ultrasonographic findings and outcome of 17 dogs with non-enteric plant material foreign bodies migration [28]. Two of seventeen dogs had grass awns in the sublumbar region, but US was able to locate the foreign body in only one dog in the second examination. The authors hypothesized that the large amount of exudate, with many scattered brightly echogenic elements, prevented visualization of the vegetal foreign bodies. Two other studies reported the limitations of ultrasonography to locate grass awn in the retroperitoneal region but supported its utility as a complementary tool to more advanced imaging techniques such as MRI and CT [21,27]. In contrast to these findings, a study published in 2017 by Birettoni and colleagues reported the usefulness of preoperative and intraoperative US for visualizing and removing plant material from iliopsoas abscesses. Grass awns typically appeared ultrasonographically as spindle-shaped, shadow casting, hyperechoic structures of variable length [10]. In all the 22 dogs included in this study, ultrasonography was successfully used to direct the forceps through the iliopsoas muscle and to guide the grass awn removal. Furthermore, the authors emphasized that in 11 of the 22 dogs, the foreign bodies had been partially removed during a previous standard surgery, suggesting that US guidance increases the likelihood of complete grass awn removal [10]. This contrasts with a retrospective study in which the recurrence rates and the number of dogs that had foreign bodies removed with laparotomy alone and laparotomy with intraoperative ultrasonography were similar. However, intraoperative ultrasonography was used only for complex cases, which might explain the findings of this study [26]. Recently, Moretti and colleagues described a novel mini celiotomy approach using a small ring retractor in dogs. This tool allowed a simultaneous insertion of both the US probe and the grasping forceps for the retrieval of migrating grass awns from the iliopsoas muscles [25]. Although iliopsoas abscesses can extend within the retroperitoneal space, kidneys and vascular structures are usually spared. A case report described a retroperitoneal pseudoaneurysm of the celiac artery in a dog associated with a migrating grass awn. In this patient, abdominal US was able to localize the lesion but failed in detecting the vegetal foreign body. Authors concluded that the ill-defined margins of the acoustic shadow within the lesion were not sufficiently definitive to differentiate gas from a foreign body [29]. An unusual location of migrating grass awn was described in a recent case report. In this dog, the vegetal foreign body caused a subcapsular renal abscess and preoperative abdominal US was able to accurately identify it [23]. Retroperitoneal migrating grass awns are rarely reported in cats. The low incidence in cats can be due to feline behavior such as extensive self-cleaning and closed-mouth breathing. Like in dogs, vegetal foreign bodies can migrate in the thoracic cavity or through the diaphragmatic crura toward the retroperitoneal space, leading to fistulous tracts and iliopsoas abscess formation [13,19,30,31]. A case report published in 2018 described a retroperitoneal abscess associated with a migrating grass awn in a domestic short hair cat [30]. Although abdominal US revealed a mixed echogenicity fluid-filled mass, it was unable to visualize the grass awn which was later found during surgical exploration [30]. In contrast, three papers underlined the high diagnostic accuracy of ultrasonography in the detection of foreign bodies in the retroperitoneal space and their safe removal under US guidance in cats [13,19,31]. As in dogs, US allowed visualization of hyperechoic structures with linear interfaces and acoustic shadowing inside the sublumbar region.

### 3.3. Intraabdominal Migration

Migration of vegetal foreign bodies in the abdominal cavity is not often reported in dogs and cats. However, it can lead to severe consequences such as septic peritonitis and pancreatitis [12,32,33]. For this reason, early diagnosis is essential. Abdominal US has been well described as a useful technique to identify foreign bodies and to plan their surgical removal [12,28,33]. In the Hopper study, abdominal US evidenced a hyperechoic foreign body with a dense acoustic shadow, consistent with a grass awn, causing a focal peritonitis medial to the descending duodenum in a working kelpie dog [12]. In this patient, the presence of initial respiratory signs led to the suspicion that the vegetal foreign body have been inhaled and then migrated from the thorax to the peritoneal cavity [12]. In another study, an omental abscess and a granuloma-like lesion were identified by US between the pancreas and the right liver lobe in one dog and between the pancreas and the right cranial abdominal wall in another. In this paper, the authors hypothesized that in both cases, the foreign bodies were inhaled and then migrated through the right diaphragmatic pillar and the liver to reach the right cranial abdominal region [33]. A spindle-shaped structure with several reflecting interfaces, consistent with a migrating vegetal foreign body, was visualized by ultrasonography within a dilated common bile duct of a vomiting cat. In this patient, the grass awn was the cause of the extrahepatic biliary obstruction [34]. On the contrary, in a study published by Culp et al., US examination was not able to detect the migrating grass awn, which was later found during the surgery in the spleen of a cat with septic peritonitis [32]. In 2019, another paper reported the lack of US visualization of a grass awn in the peritoneum of a cat in which the vegetal foreign body caused jejunal perforation. The authors speculated that the peritoneal effusion, together with the intestinal gas content, can have impeded the identification of the grass awn in the peritoneal cavity [19].

### 3.4. Ocular Region Migration

Migration of grass awns in the ocular region has been described in veterinary literature mainly in dogs and rarely in cats. The plant material usually remains in the retrobulbar area causing orbital abscess and cellulitis. Sometimes, they can migrate through the sclera and reach the posterior segment of the eye leading to severe inflammation, panophthalmitis, or secondary uveitis-associated glaucoma [35,36]. The main clinical signs include ocular discharge, exophthalmos, protrusion of the third eyelid, and ocular pain [35,37,38]. Ultrasonography is a rapid and noninvasive technique for evaluating ocular disease, but differentiation between foreign bodies and other inflammatory and/or neoplastic conditions can be challenging. A case report described the use of ocular ultrasonography in a dog with a 3-week history of right sided exophthalmos, periorbital swelling, and chemosis. The ultrasonographic examination revealed an echogenic nodule, with a hypoechoic center within the posterior tissue planes of the right globe but failed to visualize the vegetal foreign body. The authors highlighted the difficulty of establishing an etiological diagnosis using ocular ultrasonography [36]. Similarly, in another case report, ultrasonography was used to drain an abscess in the left medial temporalis muscle of a dog, but the grass awn was not visualized [39]. In two recent case reports, ocular and orbital US failed to identify the presence of grass awns in the conjunctivoscleral region [40] and in the intraconal part of the retrobulbar space [41]. In contrast with these findings, several studies demonstrated the utility of ultrasonography in diagnosing ocular foreign bodies [38,42,43]. Ultrasonography was able to identify a spindle-shaped structure with two hyperechoic interfaces, consitent with a migrating grass awn, in a palpebral abscess [38] and in the conjunctiva [42] of two dogs. Barsotti et al. reported the efficacy of US not only in diagnosis but also in removal of foreign bodies from the lacrimal sac of four dogs [43]. In cats, ocular localization of grass awns is frequent but intraocular or retrobulbar migration is rare. In 2005, Trovar et al. described an unusual migration of a vegetal foreign body through the sclera of a cat. Ocular ultrasonography revealed a suprachoroidal abscess and retinal detachment but was not able to detect the grass awn [35].

### 3.5. Genitourinary Tract Migration

**Figure 5:**
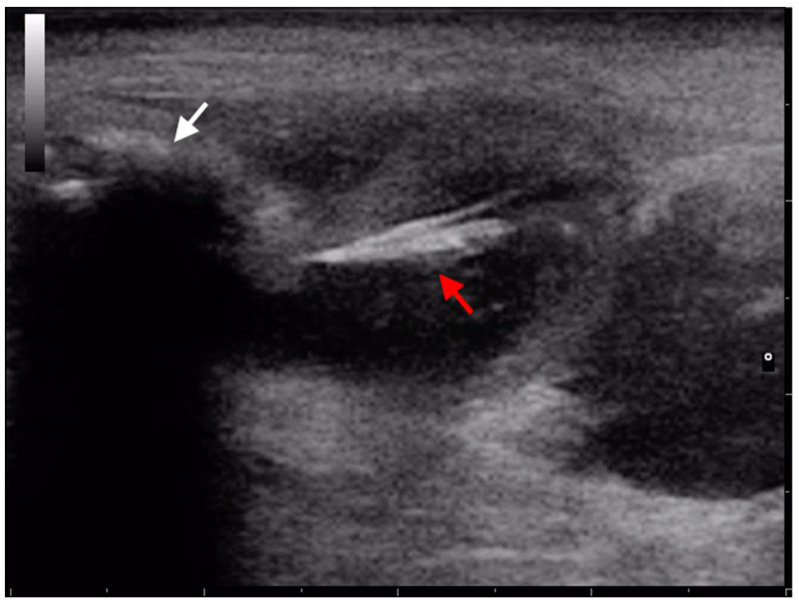
Ultrasonographic image of a spindle-shaped hyperechoic foreign body consistent with a migrating grass awn (red arrow) within the preputium of a dog. White arrow indicates the os penis.

Migration of grass awns through the genitourinary tract has been reported in dogs and cats. Penetration of grass awns into the genitourinary tract can occur by ascending migration from the vagina/urethra or, less commonly, through the skin or intestinal tract. The clinical signs include hematuria, pollakiuria, vaginal or preputial discharge [44,45,46]. Cherbinsky et al. reported the ultrasonographic visualization of intravesical migrating grass awns in three Yorkshire Terriers dogs and one cat [45]. The authors hypothesized that the penetration occurred through the penis. In one case, it was facilitated by a urethrostomy which led to dilatation of the orifice. In 3 out of 4 patients, the vegetal foreign bodies were easily identified by US and the appearance of grass awns in the bladder was consistent with their appearance in other tissues (linear or sometimes V-shaped structure casting an acoustic shadow). In a dog, the diagnosis was made after surgery because the grass awn was embedded into a vesical stone. the lesion was likely chronic based on the presence of a struvite rim around the grass awn. The authors of this study highlight the importance of including chronic bladder foreign body among the differential diagnosis when a luminal structure with a hyperechoic rim and hypoechoic center is visualized ultrasonographically. Even the presence of nodular lesions in the bladder wall requires a differential diagnosis with vegetal foreign bodies [46]. In Lomax’s study, a grass awn was visualized in a bladder wall neoformation of a miniature poodle dog. The lesion appeared as a hypoechoic nodular structure. Subsequent histological examination confirmed the presence of a suppurative inflammation of the muscular wall layer caused by a vegetal foreign body. Grass awn migration in the masculine genital apparatus was described in several studies (Figure 5) [19,47,48,49]. Del Signore et al. reported the case of a dog with a vegetal foreign body in the preputium, which was removed with Hartmann US-guided technique [48]. Bergamini et al. published a retrospective study including six dogs with hematuria and preputial discharge secondary to grass awn migration [47]. In five dogs, the vegetal foreign bodies were visualized in the balano-preputial sulcus, embedded in the subcutaneous tissue; in another dog, a grass awn was detected in the inguinal region. The authors confirmed the usefulness of US for localization and removal of migrating grass awns in all the patients [47]. In 2020, Marchesi et al. described ultrasonographic findings of a migrating grass awn in canine prostatic parenchyma and subsequent echo-guided removal of the foreign body [49]. On the contrary, in a study by Cola et al., the US technique was not supportive in diagnosing the grass awn-induced urethral obstruction and rupture in two male cats [19]. Vaginal vegetal foreign body migration has been described in dogs and cats. The most common clinical sign was vulvar discharge, which could also be associated with abdominal pain, fever, and polyuria [13,44,50,51]. In Gatel’s study, US was successfully used to guide the extraction of the vaginal foreign body in a dog and in a cat with the Hartmann forceps [51]. In 2016, Agut et al. described a case report of a urethrovaginal fistula due to vaginal grass awn. In this study, ultrasonography was able to visualize the vegetal foreign body but failed to diagnose the urethrovaginal fistula [44].

### 3.6. Subcutaneous Tissue Migration

**Figure 6:**
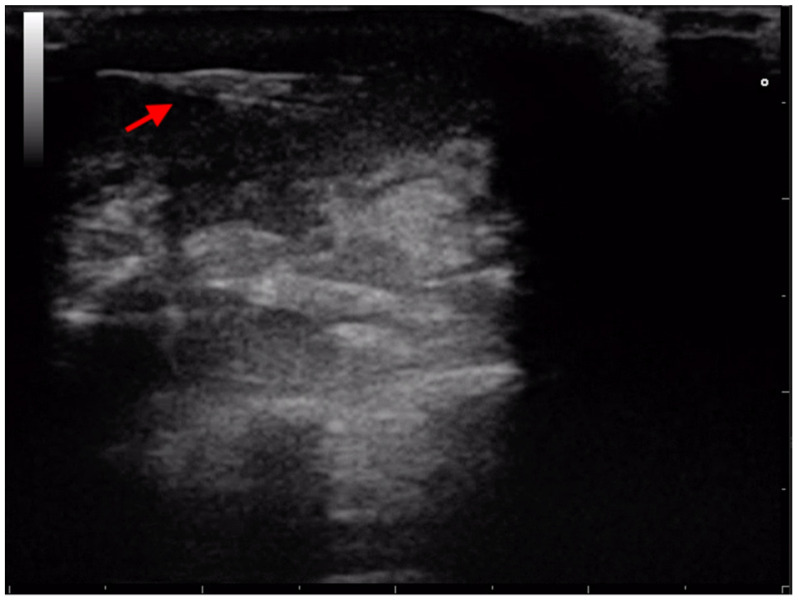
Ultrasonographic image of a spindle-shaped hyperechoic foreign body consistent with a migrating grass awn (red arrow) located in the subcutaneous space of a dog.

Migration of the grass awns in the subcutaneous and underlying soft tissues structures is common in dogs. The diagnosis can be challenging because the foreign bodies cannot be recognized on initial physical examination. Moreover, when localized, they can be incompletely removed, causing abscesses or draining tracts weeks to months later [3,8,52]. Diagnostic imaging can help clinicians to visualize and remove grass awns. A retrospective case series of six dogs described the application of ultrasonography for localization of subcutaneous foreign bodies and compared this technique with radiography and contrast fistulography. A hyperechoic structure compatible with foreign body was identified in 5 out of 6 dogs, whereas radiography was not able to detect it in any patients. The authors concluded that US is an excellent method for identification of nonradiopaque foreign bodies in dogs with chronic draining tracts/abscesses and provided useful information about the extent of the disease prior to surgery [3]. Several reports confirmed the importance of ultrasonography not only in the diagnosis (Figure 6) but also in the removal of grass awns retained in subcutaneous tissues [8,13,31,53,54,55]. Some studies reported the US-guided surgical removal of vegetal foreign bodies [8], whereas others emphasized the superiority of the nonsurgical US-guided approach. Della Santa et al. described a minimally invasive US-guided foreign body retrieval in six dogs with abscesses. All procedures were performed in the sonography room inserting the Hartmann forceps through the draining tract. When draining tracts were not present, a small skin incision was made close to the ultrasonographic location of the grass awn. The incision had to be just large enough for Hartmann forceps to be inserted and ultrasonography allowed to follow the forceps during the removal of the grass awn. The authors concluded that this minimally invasive procedure should be preferred to standard surgical retrieval since it is less invasive, readily available, and cheaper [52]. Similarly, Fauchon et al. reported a 100% success rate of US-guide removal of grass awn from the distal extremities of 19 dogs [53]. Baudin Tréhiou et al. reported the identification and removal of grass awns from thoracic wall abscess after unsuccessful thoracic surgery in two dogs [56]. In 2019, Cola et al. described the use of intraoperative ultrasonography to facilitate the location and removal of grass awn in the region of the neck, close to the cervical trachea, in a cat [19]. Recently, a case report described an unusual presentation of a migrating grass awn in a German pointer dog with lethargy, pyrexia, and swelling on the calvarium. Focal US of the left temporalis muscle region successfully identified an abscess with a vegetal foreign body within it [55].

## 4. Discussion

In the present review, we have summarized the current knowledge on the application of US in detecting and removing migrating grass awns in dogs and cats. As reported, ultrasonography is a valid diagnostic tool for visualization of migrating vegetal foreign body in various body tissues and cavities. Moreover, ultrasonography can be useful for guiding surgical removal of vegetal foreign bodies in different tissues.

The ultrasonographic appearance of grass awns is characterized by a spindle-shaped hyperechoic structure casting an acoustic shadow and surrounded by a hypoechoic area. The visualization of the grass awns can be more difficult when migration occurs in pleural space because of the presence of scattered hyperechoic elements (fibrin, tissue remnants, cellular debris, or air). Moreover, the large field that needs to be examined in these cases can limit the visualization of foreign bodies. Conversely, when intrapulmonary migration occurs, pulmonary consolidation can help the operator visualize the foreign body. To improve the visualization of migrating grass awn localized in the medial portions of the lung lobes and near the esophagus, transesophageal ultrasound should be considered [16]. Ultrasonographic visualization of grass awns can be easy if migration occurs within the iliopsoas abscesses in dogs and cats: enlargement of the affected iliopsoas muscle and unstructured hypoechoic appearance with anechoic areas of variable size and number represent characteristic US findings in sublumbar migration. Hyperechoic structures, consistent with grass awns, are frequently surrounded by an anechoic area and their identification is enhanced by this surrounding hypoechoic region of fluid associated with an inflammatory response. Peritoneal localization of migrating grass awns occurs less frequently in dogs and cats, and ultrasonographic visualization of these has been reported but with a lower accuracy than iliopsoas muscles localization. Similarly, ultrasonographic examination in dogs and cats suspected to have ocular migration of grass awns can be useful to add information, although the visualization of the foreign body could fail. Ultrasonographic examination of the subcutaneous tissue and genitourinary tract is recommended, if migrating grass awns are suspected.

Commonly, microconvex and linear probes are used to visualize migrating grass awns in various tissues. The choice depends on the depth of the lesions: lower-frequency transducers as the microconvex probes are needed for deep lesions, whereas high-frequency linear transducers are useful for superficial lesions. Moreover, microconvex probes can be useful if multiple scans are needed to identify the grass awn in body’s areas with limited access acoustic windows (i.e., intercostal spaces).

Ultrasound is a safe, readily available, economical, and minimally invasive procedure that allows to visualize the foreign body and plan its surgical removal in various body tissues and cavities in dogs and cats; moreover, intraoperative ultrasonography improves the success of the grass awn removal. However, this diagnostic tool is highly dependent upon the skills and experience of the operator and the patient size that can limit the visualization of grass awns [27,30]. Some authors have reported the benefit of combining computed tomography and ultrasound in complex cases for a higher diagnostic success, especially in identifying discrete foreign bodies [27].

## 5. Conclusions

The scientific literature examined shows that US is a valid diagnostic tool for visualization and guiding the removal of migrating grass awns in dogs and cats. The constant technological improvement of the equipment increased the quality of images and, consequently, the accuracy in detecting migrating grass awns in various body tissues and cavities. In our opinion, US can be a useful diagnostic tool to localize and remove migrating grass awns; in complex cases, where the migration occurs in less accessible locations, this diagnostic tool should be considered as a part of a multidisciplinary approach with advanced diagnostic imaging modalities.

## Data Availability

Not applicable.
